# Conduction Disorders: The Achilles’ Heel of TAVR?

**DOI:** 10.3390/jcm15031096

**Published:** 2026-01-30

**Authors:** Antonin Fournier, Pierre Robert, Jean-Christophe Macia, Jean-Michel Berdeu, Laurent Schmutz, Matthieu Steinecker, François Roubille, Guillaume Cayla, Florence Leclercq

**Affiliations:** 1 Department of Cardiology, CHU Montpellier, Arnaud de Villeneuve Hospital, University of Montpellier, Avenue du doyen Giraud, 34295 Montpellier, France; antonin.fournier@hotmail.fr (A.F.); jc-macia@chu-montpellier.fr (J.-C.M.); jmberdeu34@gmail.com (J.-M.B.); matthieu.steinecker@chu-montpellier.fr (M.S.); francois.roubille@gmail.com (F.R.); 2 Department of Cardiology, CHU Nîmes, 30029 Nimes, France; pierrecardio@gmail.com (P.R.); laurent.schmutz@chu-nimes.fr (L.S.); cayla.guillaume@gmail.com (G.C.)

**Keywords:** TAVR, ECG, conductive disorders, permanent pacing

## Abstract

High degree conductive disorders (CD) requiring permanent pacemaker implantation (PPI) have modestly decreased over time and remain the main complications of TAVR. Furthermore, management strategies for CD occurring after TAVR remain controversial. We proposed a review evaluating mechanisms and risk of CD after TAVR, focusing on the role of ECG evaluation but also on the importance of anatomic parameters analyzed in multi-slice computed tomography (MSCT), as well as regarding procedural aspects. Considering the lack of clear recommendations for the evaluation of risk of CD and indications of PPI, this review tries to summarize strategies to anticipate and detect the risk of high degree CD, to decrease incidence of CD and to optimize PPI indications. Perspectives regarding ambulatory monitoring, use of machine learning and new pacing techniques are proposed. This review was narrative and included selection of literature using key words including: conductive disorders, TAVR and pacemaker implantation.

## 1. Introduction

**Figure 1:**
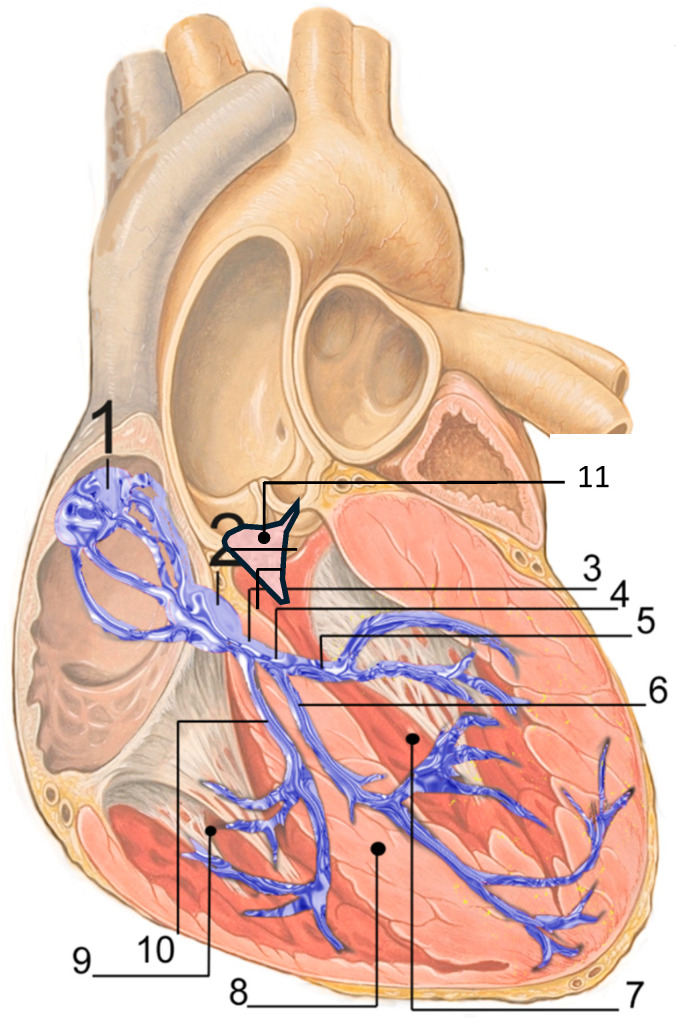
Cardiac conduction system by J. Heuser, licensed under CC BY 2.5/Wikimedia Commons. URL: https://commons.wikimedia.org/w/index.php?title=File:RLS_12blauLeg.png&oldid=971131153 (accessed on 10 December 2025). 1. Sinoatrial node; 2. Atrioventricular node; 3. Bundle of His; 4. Left bundle branch; 5. Left posterior fascicle; 6. Left-anterior fascicle; 7. Left ventricle; 8. Ventricular septum; 9. Right ventricle; 10. Right bundle branch; 11. Membranous septum.

Transcatheter aortic valve replacement (TAVR) may expose the conduction system (Figure 1) to both ischemic and mechanical injury in conjunction with a subacute healing process that may contribute to severe conduction disturbances (CD), occurring not only acutely but also, although less frequently, at later stages, including high degree atrioventricular block (HAVB) and complete atrioventricular block (CHB) [1]. CD occurring after TAVR are a critical challenge. They remain the main complications of the procedure and despite both improvement of devices and experience of operators, permanent pacemaker implantation (PPI) has modestly declined over time, remaining at between 7 to 22% according to studies and devices [1,2], Table 1. Furthermore, CD contribute largely to prolonged hospitalization after TAVR [3,4].

A recent survey of the European Heart Rhythm Association (EHRA) revealed significant heterogeneity in CD management post-TAVR across European centers [5]. While ECG telemetry is commonly used for patient monitoring, protocols regarding the timing and indication of PPI differ significantly, leading to notable discrepancies in implantation rates among centers. For example, a standardized management protocol for advanced conduction disorders such as LBBB or atrioventricular block (AVB) was available in only 63% of participating centers and the duration of telemetry in patients with new-onset LBBB varied from a 48 h to at least a 72 h period. New-onset LBBB and PPI during TAVR are linked to unfavorable long-term clinical outcomes, such as hospitalization for heart failure and death, whereas this causality remained debated considering pacing burden and underlying disease [6]. As TAVR is used in younger and lower-risk populations, these clinical outcomes are going to become more significant [6,7]. Recent consensus documents emphasize a comprehensive approach that includes pre-procedure risk assessment, intraoperative strategies, and post-TAVR monitoring to minimize conduction disturbances and improve patient outcomes [8,9].

The evolution of TAVR toward a minimalist procedure with shorter hospital stay (generally 1–2 days) has further underscored the importance of effective CD management. However, this shift has also been associated with a rise in readmission rates due to delayed bradyarrhythmia occurring more than 24–48 h post-procedure [10]. On the other hand, with the decrease of hospitalization length after TAVR, the risk is to implant pacemakers in lower degrees of CD in order to decrease ICU or monitoring care has to be considered. The challenge lies in balancing fast-track protocols with ensuring patient safety.

This review proposes:

- To evaluate the risk of high degree CD after TAVR using ECG and non-ECG parameters and, particularly, the impact of CT scan evaluation and device positioning
- To propose management of CD including indications and length of telemetry monitoring and pacemaker indications regarding literature data. Questions regarding electrophysiology evaluation or ambulatory ECG monitoring were discussed.
- To give emergent data and perspectives regarding particularly connected wearables for monitoring, left bundle branch stimulation, dual chamber leadless pacemaker or machine learning for risk prediction.

### 1.1. Evaluation of the Risk of High Degree CD

#### 1.1.1. ECG Analysis

**Pre-procedural assessment** of the risk of severe CD requiring PPI has been largely documented in several studies. Key factors influencing this risk include:

- Pre-existing right bundle branch block (RBBB), which is present in almost 10% of patients undergoing TAVR and is associated with a three-to-four-fold increased risk of PPI at 30 days [11,12], with a PPI rate exceeding 25% in this subgroup [8].
- First-degree atrioventricular block (AVB), which approximately doubles the risk of PPI at 30 days [13].
- Pre-procedural LBBB or left anterior hemiblock, which have also been identified as risk factors for HAVB or CHB as well as PPI post-TAVR [6,14].
- Episodes of HAVB/CHB or severe bradycardia diagnosed by 24 h continuous ECG monitoring before the TAVR procedure, which were reported by Urena et al. as associated with PPI required in one-third of patients; but the feasibility and generalizability of routine 24-h monitoring before TAVR in contemporary practice should be considered [15].

##### During and After TAVR

Based on ECG changes or arrhythmias observed during and after the procedure and the analysis of the ECG before TAVR, patients may exhibit either no ECG changes or develop new conduction disturbances.

New-onset LBBB persisting at the end of the procedure remains the most frequent CD, occurring in approximately 25% of patients. It has been identified as a risk factor for periprocedural PPI and was more frequently observed with the first-generation CoreValve system [1]. LBBB causes ventricular desynchronization and remodeling, which can lead to impaired LVEF and promote the onset of arrhythmias. Its impact on major cardiovascular events and mortality has been controversial in the literature [7,16,17,18,19,20], but recent meta-analyses have shown that it is associated with long-term excess mortality [21,22].

New onset LBBB with a long QRS duration (>150 ms, regardless of PR interval) and first-degree AVB (PR > 240 ms) have been associated with an increased risk of delayed HAVB/CHB and sudden death [7]. Longer QRS duration may be associated with more fibrosis and left ventricular dysfunction [6,7].

Previous studies have shown that approximately one third of patients with a typical left bundle branch block (LBBB) morphology on surface ECG exhibit normal or near-normal ventricular electrical propagation patterns [23]. Therefore, the presence of LBBB on ECG does not necessarily imply significant mechanical dyssynchrony. This electrophysiological and mechanical heterogeneity may partly explain the variability in prognosis associated with LBBB reported in the literature. A recent study showed that surface ECG alone poorly reflects underlying conduction system damage after TAVI. QRS or PR prolongation may not reliably indicate infra-Hissien delay, which is better captured by HV measurement [24].

Procedural or persistent HAVB/CHB occurs in approximately 5% of TAVR procedures and is strongly associated with early PPI in the majority of patients [25]. Importantly, most HAVB/CHB episodes develop during the procedure itself, with fewer than 20% occurring during the post-procedural period [26,27]. This observation supports a more selective approach to post TAVR monitoring, providing a rationale for avoiding excessive hospitalization length post procedure, which could be justified by the possible occurrence of delayed HAVB/CHB. Importantly, the vast majority of delayed (post-procedural) HAVB/CHB episodes occur in patients with either pre-existing or new-onset conduction disturbances with the main risk factor associated with preprocedural RBBB (OR: 4) [27]. In all studies and despite few direct comparative studies, self-expandable THVs, particularly intra annular devices, were associated with a higher risk of CD and PPI rates than balloon-expandable devices (Table 1).

The need of patient monitoring by telemetry is a tradeoff with early mobilization and timely discharge. Intensive Care Unit admission should not be always considered for monitoring in asymptomatic CD, and, when necessary, the temporary pacemaker should be inserted via a jugular approach, allowing the patient to be mobilized as soon as possible.

#### 1.1.2. Anatomical Considerations via CT Imaging

- Membranous septum (MS) length

**Figure 2:**
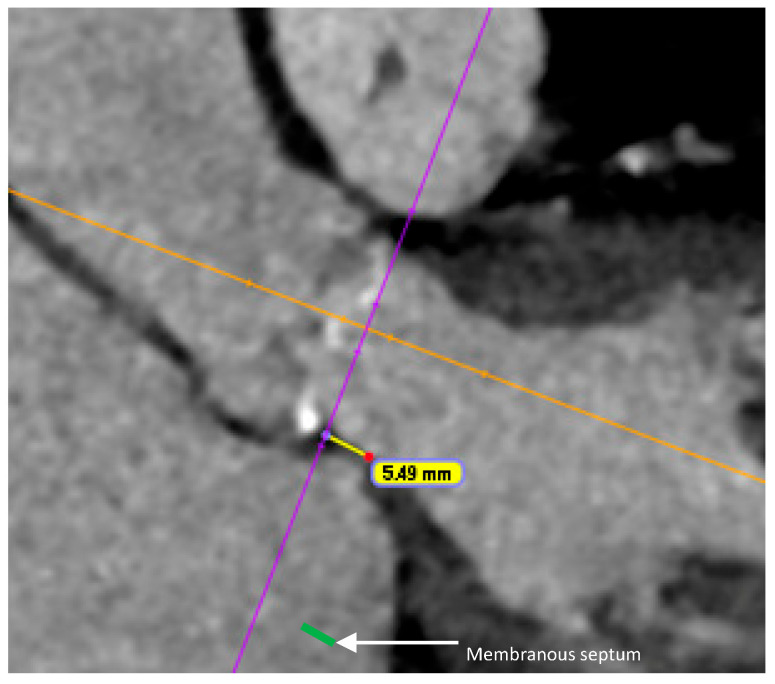
Length of membranous septum (5.49 mm) measured on coronal view of CT scan.

A shorter MS has been identified as an independent predictor of new-onset CD post-TAVR. Mechanical compression of the conduction tissue is more likely when the MS is short, particularly if the difference between the MS length and the implantation depth is insufficient [28,29] (Figure 2). A cutoff of 6–7 mm [30], as well as an implantation depth exceeding 70% of the MS length, has been strongly associated with CD post TAVR [31]. Pre-procedural planning with accurate measurement of the MS length on CT scan is essential to determine the optimal implantation depth and minimize the risk of compressing the conduction tissue. Interobserver variability, different methods of measurement and reproducibility of MS measurement in routine CT workflows have to be pointed out, however, and may limit the study comparisons. Furthermore optimal thresholds may likely vary by valve platform [28,29].

- Calcifications

Both the volume and distribution of valvular and left ventricular outflow tract (LVOT) calcification have been reported as factors that increase the risk of severe CD [1,32,33]. Several studies assessing the risk for conduction disturbances after TAVR have pointed to the importance of anatomic circumstances like large aortic valve dimensions, calcification patterns of the device landing zone, and surrounding structures potentially affected by mechanical compression during valve deployment. Elevated calcium load of the left coronary cusp and of the non-coronary cusp could be identified as predictors for PPI after TAVR but also total LVOT calcium volume [28]

Further prospective studies including these parameters are needed to confirm their prognostic impact in predicting post-TAVR CD.

#### 1.1.3. Device Selection

The type of transcatheter heart valve (THV) may impact the risk of CD. First-generation self-expandable valves have been associated with higher PPI rates, due to a more pronounced mechanical injury to the conduction system, continuous radial forces, deeper implantation and more tissue edema compared with balloon-expandable valves [8]. Conversely the new generation balloon-expandable Sapien 3 valve frame has greater height than the XT, which may extend deeper into the LVOT after deployment, and a skirt for the Sapien 3 Ultra, which leads to an increased risk of CD [34,35]. Despite a reduction in PPI rates with the new Evolut PRO/PRO+, the rates remained significantly higher compared to the balloon-expandable devices [2]. In the intra-annular self-expandable (Abbott, Chicago, IL, USA) a high incidence of PPI was described with a rate of 19% at 30 days [36].

A low incidence of PPI has been reported in Acurate Neo implantation with an overall rate of 8.3% in a large cohort [37]. However, the device has recently been withdrawn from the market, following the release of data showing the devices were linked to higher rates of all-cause death, stroke, and rehospitalization than were other commercially available valves [38].

**Table 1:** PPI rate at 1-month by THV type.

THV	PPI Rate Range	References
**Accurate Neo**	**8–15%**	Möllmann [37]: 8.3%
		Wang [2]: 9.7%
		Husser [39]: 9.9%
		Costa [40]: 14.7%
**S3/S3 ultra**	**7–15%**	Mack [41]: 6.5%
		Wang [2]: 11.5%
		Costa [40]: 12.5%
		Husser [39]: 15.5%
**Evolut R/pro**	**16–20%**	Wang [2]: 16.9%
		Popma [42]: 17.4%
		Costa [40] 19.3%
**Portico/Navitor**	**17–22%**	Garcia [43]: 16.6%
		Reardon [36]: 19%
		Costa [40] 22.1%

#### 1.1.4. Valve Implantation Technique/Implantation Depth

Prior studies have demonstrated the influence of THV position on the occurrence of new CD and paravalvular leakage (PVL) in self-expandable but also in balloon-expandable devices

- The “cusp overlap” technique (COT)

This periprocedural technique involves a change in implant projection, applying an overlap projection of the left coronary cusp (LCC) and right coronary cusp (RCC), while isolating the non-coronary cusp (NCC). This technique developed originally with the self-expandable Medtronic device, reduces parallax, allows for a more accurate assessment of the true device implantation depth and minimizes LVOT contact by starting the valve deployment from above the aortic annulus [44,45,46]. The COT also achieves a proper coronary/commissural alignment to facilitate coronary cannulation, which is particularly important in self-expandable THVs but seems also to facilitate more accurate THV positioning with balloon-expandable devices.

This technique may help reduce valve implantation depth, thereby minimizing interference with the conduction system [47]. However, we have to keep in mind that higher implantation may complicate future coronary access or potentially compromise the feasibility of redo-TAVR whereas commissural alignment may be considered as a mitigating strategy [48].

- Implantation depth is one of the most modifiable procedural predictors of PPI across different THV platforms [34,49,50]. Depth greater than 5–7 mm below the aortic annulus has been associated with an increased risk of new-onset LBBB [8].
- The MIDAS technique—the MInimizing Depth According to the membranous Septum (MIDAS) technique—has been shown to decrease PPI rates with self-expanding valves [51]. Implantation depth greater than MS length was independently associated with PPI with a self-expanding THV, highlighting the utility of MS length-based implantation strategies. It is a patient-specific, anatomy-based approach that is easy to apply but which has to be validated in a multicenter study.
- Oversizing:

In exchange for preventing PVL, a significant degree of oversizing regarding annular size may increase the risk of CD after TAVR [14,52]. Although data are limited in patients with borderline annulus sizing, the selection of a smaller or a larger THV remains sometimes uncertain. Minimal oversizing, ranging from 0% to 5%, has been reported to be optimal for the S3 ultra THV, balancing the risk of PVL and CD [53]. Particular caution with oversizing should be the rule in case of prior CD in patients undergoing TAVR (Table 2).

### 1.2. CD Management

The management of TAVR-related CD during the post-procedural period remains controversial and highly variable across centers.

#### 1.2.1. Temporary Pacing

Transvenous pacing wires allow rapid ventricular pacing and facilitate optimal valve implantation. This requires additional venous access, with the inherent risk of vascular complications. In addition, temporary pacing lead carries the risk of right ventricle (RV) perforation and pericardial effusion. Lead instability in the RV can result in loss of capture and valve embolization. Alternatively, pacing via the left ventricular (LV) valve delivery guidewire avoids these complications, provides rapid and stable pacing and simplifies the procedure [54].

For patients at high risk of CD (e.g., prior RBBB or AVB), LV pacing is however less attractive as pacing may not be effective after valve delivery catheter retrieval. According to recent consensus, in cases of prior RBBB or development of CD (such as new LBBB or CHB), a temporary RV pacing lead is recommended before the patient leaves the procedure room for at least 24 h [8].

#### 1.2.2. Telemetry Monitoring (TM)

It is common practice to monitor all patients for at least 24 h after TAVR [8]. Extended monitoring is recommended in patients who develop CD to evaluate their evolution (regression, stabilization or progression).

For patients developing wide LBBB or LBBB associated with first degree AVB with PR interval > 240 ms, ESC guidelines recommend extended outpatient monitoring for 14 days [55].

Some studies indicate that patients with normal ECG or stable CD may not require systematic TM (approximately 1/3 of patients). In fact, most patients undergoing TM did not experience any rhythmic events, highlighting the need for better patient selection [4].

Our team recently assessed the safety of a selective TM approach for patients with preexisting RBBB, intraprocedural AVB and new or worsening LBBB and developed an algorithm to guide ICU indication and length of stay [56].

#### 1.2.3. Electrophysiology Study (EPS)

EPS may help determine the need for PPI, particularly in patients with new-onset LBBB. However, variability in EPS protocols across centers underscores the need for standardization. The EHRA suggests that an HV interval > 75 ms during EPS should prompt PPI [5]. For patients without a baseline pre-TAVR RBBB or without new persistent post-TAVR LBBB, an HV ≤ 65 ms was described as completely ruling out the incidence of high degree AVB.

Selective EPS can be considered in patients with borderline PPI indications, given the limitations of currently available data [57]. The randomized MONITOR TAVI trial will provide further insights into the role of EPS in CD management after TAVR [NCT 06148883].

An alternative approach is to measure the HV interval using a temporary pacemaker wire: patients with post-TAVR LBBB and an HV interval < 55 ms are considered at low risk for developing CHB with 90% negative predictive value [58].

#### 1.2.4. Pacemaker Indications

Attitudes towards PPI indications post-TAVR vary considerably between centers. The 2021 ESC Guidelines recommend PPI if intraprocedural complete or high-grade AVB persists for 24–48 h post-TAVR (class I) or in the case of alternating bundle branch block (class I) or prior RBBB associated with another conduction abnormality occurring post-TAVR (class IIa) [55]. However, in a context of shorter hospital stays, PPI indications could be more liberal to prevent high degree CD and facilitate faster discharge from the hospital, whereas concern for delayed severe CD may also unnecessarily prolong hospitalization. This is well illustrated by high discrepancies in PPI rates between studies, particularly after LBBB [5,6,8,9].

#### 1.2.5. Pacemaker Dependency/Stimulation Rate

Studies have shown that, regardless of the THV implanted, only 40% to 70% of patients with new PPI following TAVR are pacemaker-dependent at one year [52,59]. This supports a more conservative approach to PPI, given that a significant percentage of patients recover conduction function over time. However, the ideal degree of pacemaker dependency during TAVR has not been established. Even minimal pacing may be lifesaving in case of CHB, despite low stimulation rates. Optimal programming of devices to limit unnecessary pacing may be also be considered.

#### 1.2.6. Left Bundle Branch Area Pacing (LBBAP)

LBBAP is gaining interest for providing more physiological pacing, especially in patients with LBBB and reduced left ventricular ejection fraction (LVEF) post-TAVR. Studies show LBBAP results in significantly shorter paced QRS duration, greater improvement in LVEF over time and lower risk of heart failure rehospitalization at five years compared with right ventricular pacing [60]. In patients with reduced LVEF, cardiac resynchronization therapy or other physiological pacing should be considered prior to TAVR. Caution should, however, be emphasized regarding operator expertise and lead placement challenges in post-TAVR anatomy.

#### 1.2.7. Ambulatory ECG Monitoring (AEM) and Late Conductive Disorders

In the context of early discharge protocols, AEM has emerged as a valuable tool for detecting delayed CD, especially in the first month post-TAVR. Implementing AEM may help to prevent late cardiac syncope or death due to post-discharge HAVB, as well as avoiding unnecessarily early PPI.

Ream et al. identified delayed HAVB requiring PPI in 8% of cases. The MARE study showed that 20% of patients experienced severe episodes of bradyarrhythmia, 76% of which were asymptomatic [61]. The REDIRECT TAVI study further underscores the utility of AEM both pre- and post-TAVR, improving detection of CD requiring PPI and contributing to shorter hospital stays and lower PPI rates [62].

However, current data supports the use of AEM in selected patients (baseline RBBB, new-onset LBBB, transient CHB) [9,55].

### 1.3. Emerging Trends

#### 1.3.1. Connected Wearables for Monitoring

Connected smartwatches with ECG and heart rate monitoring provide a non-invasive, high adoption and affordable method to detect transient or intermittent CD. This continuous monitoring may facilitate safer outpatient monitoring in the near future.

A single-center study evaluated the efficacy of smartwatch ECG monitoring in detecting arrhythmic events within 30 days post-TAVR, showing acceptable diagnostic performance for identifying atrial fibrillation, LBBB and RBBB [63]. The SMART TAVR trial assessed smartwatches recording single-lead ECG with transmission to a dedicated smartphone application, which were analyzed by physicians at a central ECG core laboratory [64]. Integrating smartwatch technology into post-TAVR care protocols offers a non-invasive efficient method for continuous cardiac monitoring, facilitating early detection and management of arrhythmic events. We, however, need selective criteria to propose these systems. Furthermore, limitations related to false positives and data overload should be acknowledged.

#### 1.3.2. Machine Learning for Risk Prediction

Machine learning is increasingly being integrated into the management CD after TAVR.

Traditional risk models, such as the D-PACE score, based on procedural features (valve-in-valve, self-expandable valve), ECG and MS length, have been shown to discriminate patients at risk for delayed AVB and could be helpful in identifying patients eligible for early discharge [65]. Building upon such conventional approaches, artificial intelligence has recently been applied to analyze pre-TAVR ECGs to identify patients at higher risk of post-procedural arrhythmic events in a single-center retrospective study, and it seems promising [66]. Confirmation in prospective and multicenter studies is however required to confirm clinical impact.

#### 1.3.3. Dual Chamber Leadless Pacemaker

This new mode of pacing represents an important advancement in cardiology care, eliminating several risks associated with transvenous pacemakers. Leadless pacemakers also offer cosmetic advantages, avoiding visible incisions or bumps on the skin. They can be removed and reimplanted easily when the battery is depleted. No issues with airport security or MRI are observed. Leadless pacemakers are increasingly being used for CD following TAVR and were associated with a lower rate of in-hospital and midterm device-related complications compared to transvenous pacemakers without a difference in midterm mortality [67]. Current limitations are related to the absence of dual-chamber synchronization when required and issues regarding retrieval in long-term follow-up.

## 2. Conclusions

Despite improvements in devices and procedural techniques, experienced operators and a lower risk profile in the population, CD remain a major issue after TAVR. Both the indications and duration of TM as well as the criteria for PPI vary widely across centers in the absence of clear recommendations. Considering shorter hospitalization post-TAVR, there is a risk of implanting pacemakers in order to reduce ICU or monitoring care. The objective of shorter hospitalization length must not be obtained at the expense of safety. Recent studies have shown that selective TM post-TAVR is possible without compromising safety. Future studies should aim to rationalize PPI indications during hospitalization and accurately anticipate the risk of late CD. Multiparameter strategies integrating ECG analysis, procedural factors and anatomic characteristics, potentially enhanced by machine learning, may help achieve this goal. Specific research priorities may include prevention of CD with new devices or implantation techniques, randomized monitoring strategies and new pacing approaches.

## Figures and Tables

**Table 2:** Advantages and disadvantages of TAVI implantation techniques.

Technique	+	−
**Cusp overlap and MIDAS technique**	▪ Allows higher and more controlled implantation with self-expanding valves ▪ Reduces the risk of CD and PPI	▪ Compromise future coronary access ▪ Risk of coronary occlusion during redo TAVR. ▪ Risk of embolization into aorta ▪ Learning curve
**Oversizing**	▪ Reduce paravalvular leak risk ▪ Better hemodynamic performance	▪ Increase PPI risk ▪ Risk of annular rupture

CD: conductive disorder; PPI: permanent pacemaker implantation.

## Data Availability

Data sharing is not applicable (the article describes entirely theoretical research).

## References

[1] Auffret V., Puri R., Urena M., Chamandi C., Rodriguez-Gabella T., Philippon F., Rodés-Cabau J. (2017). Conduction Disturbances After Transcatheter Aortic Valve Replacement: Current Status and Future Perspectives. Circulation.

[2] Wang B., Mei Z., Ge X., Li Y., Zhou Q., Meng X., An G. (2023). Comparison of outcomes of self-expanding versus balloon-expandable valves for transcatheter aortic valve replacement: A meta-analysis of randomized and propensity-matched studies. BMC Cardiovasc. Disord..

[3] Barbe T., Levesque T., Tron C., Hemery T., Bouhzam N., Bettinger N., Chaumont C., Anselme F., Eltchaninoff H., Durand E. (2022). Impact of conductive disturbances on length of stay after TAVI: A single-centre retrospective study. Arch. Cardiovasc. Dis. Suppl..

[4] Akodad M., Aldhaheri E., Marin G., Roubille F., Macia J.-C., Gandet T., Delseny D., Schmutz L., Lattuca B., Robert P. (2021). Transcatheter aortic valve replacement performed with selective telemetry monitoring: A prospective study. Int. J. Cardiol..

[5] Badertscher P., Knecht S., Zeljković I., Sticherling C., de Asmundis C., Conte G., Barra S., Jedrzej K., Kühne M., Boveda S. (2022). Management of conduction disorders after transcatheter aortic valve implantation: Results of the EHRA survey. Europace.

[6] Fischer Q., Himbert D., Webb J.G., Eltchaninoff H., Muñoz-García A.J., Tamburino C., Nombela-Franco L., Nietlispach F., Moris C., Ruel M. (2018). Impact of Preexisting Left Bundle Branch Block in Transcatheter Aortic Valve Replacement Recipients. Circ. Cardiovasc. Interv..

[7] Urena M., Webb J.G., Eltchaninoff H., Muñoz-García A.J., Bouleti C., Tamburino C., Nombela-Franco L., Nietlispach F., Moris C., Ruel M. (2015). Late cardiac death in patients undergoing transcatheter aortic valve replacement: Incidence and predictors of advanced heart failure and sudden cardiac death. J. Am. Coll. Cardiol..

[8] Rodés-Cabau J., Ellenbogen K.A., Krahn A.D., Latib A., Mack M., Mittal S., Muntané-Carol G., Nazif T.M., Sondergaard L., Urena M. (2019). Management of Conduction Disturbances Associated with Transcatheter Aortic Valve Replacement: JACC Scientific Expert Panel. J. Am. Coll. Cardiol..

[9] Lilly S.M., Deshmukh A.J., Epstein A.E., Ricciardi M.J., Shreenivas S., Velagapudi P., Wyman J.F. (2020). 2020 ACC Expert Consensus Decision Pathway on Management of Conduction Disturbances in Patients Undergoing Transcatheter Aortic Valve Replacement: A Report of the American College of Cardiology Solution Set Oversight Committee. J. Am. Coll. Cardiol..

[10] Mazzella A.J., Hendrickson M.J., Arora S., Sanders M., Li Q., Vavalle J.P., Gehi A.K. (2021). Shifting Trends in Timing of Pacemaker Implantation After Transcatheter Aortic Valve Replacement. JACC Cardiovasc. Interv..

[11] Auffret V., Webb J.G., Eltchaninoff H., Muñoz-García A.J., Himbert D., Tamburino C., Nombela-Franco L., Nietlispach F., Morís C., Ruel M. (2017). Clinical Impact of Baseline Right Bundle Branch Block in Patients Undergoing Transcatheter Aortic Valve Replacement. JACC Cardiovasc. Interv..

[12] Watanabe Y., Kozuma K., Hioki H., Kawashima H., Nara Y., Kataoka A., Nagura F., Nakashima M., Shirai S., Tada N. (2016). Pre-Existing Right Bundle Branch Block Increases Risk for Death After Transcatheter Aortic Valve Replacement with a Balloon-Expandable Valve. JACC Cardiovasc. Interv..

[13] Sammour Y., Sato K., Kumar A., Gajulapalli R.D., Lak H., Chawla S., Banerjee K., Kaur M., Patel J., Incognito C. (2021). Impact of baseline conduction abnormalities on outcomes after transcatheter aortic valve replacement with SAPIEN-3. Catheter. Cardiovasc. Interv..

[14] Siontis G.C.M., Jüni P., Pilgrim T., Stortecky S., Büllesfeld L., Meier B., Wenaweser P., Windecker S. (2014). Predictors of Permanent Pacemaker Implantation in Patients with Severe Aortic Stenosis Undergoing TAVR. J. Am. Coll. Cardiol..

[15] Urena M., Hayek S., Cheema A.N., Serra V., Amat-Santos I.J., Nombela-Franco L., Ribeiro H.B., Allende R., Paradis J.-M., Dumont E. (2015). Arrhythmia burden in elderly patients with severe aortic stenosis as determined by continuous electrocardiographic recording: Toward a better understanding of arrhythmic events after transcatheter aortic valve replacement. Circulation.

[16] Testa L., Latib A., De Marco F., De Carlo M., Agnifili M., Latini R.A., Petronio A.S., Ettori F., Poli A., De Servi S. (2013). Clinical Impact of Persistent Left Bundle-Branch Block After Transcatheter Aortic Valve Implantation with CoreValve Revalving System. Circulation.

[17] Regueiro A., Abdul-Jawad Altisent O., Del Trigo M., Campelo-Parada F., Puri R., Urena M., Philippon F., Rodés-Cabau J. (2016). Impact of New-Onset Left Bundle Branch Block and Periprocedural Permanent Pacemaker Implantation on Clinical Outcomes in Patients Undergoing Transcatheter Aortic Valve Replacement. Circ. Cardiovasc. Interv..

[18] Nazif T.M., Williams M.R., Hahn R.T., Kapadia S., Babaliaros V., Rodés-Cabau J., Szeto W.Y., Jilaihawi H., Fearon W.F., Dvir D. (2014). Clinical implications of new-onset left bundle branch block after transcatheter aortic valve replacement: Analysis of the PARTNER experience. Eur. Heart J..

[19] Chamandi C., Barbanti M., Munoz-Garcia A., Latib A., Nombela-Franco L., Gutiérrez-Ibanez E., Veiga-Fernandez G., Cheema A.N., Cruz-Gonzalez I., Serra V. (2019). Long-Term Outcomes in Patients with New-Onset Persistent Left Bundle Branch Block Following TAVR. JACC Cardiovasc. Interv..

[20] Nazif T.M., Chen S., George I., Dizon J.M., Hahn R.T., Crowley A., Alu M.C., Babaliaros V., Thourani V.H., Herrmann H.C. (2019). New-onset left bundle branch block after transcatheter aortic valve replacement is associated with adverse long-term clinical outcomes in intermediate-risk patients: An analysis from the PARTNER II trial. Eur. Heart J..

[21] Faroux L., Chen S., Muntané-Carol G., Regueiro A., Philippon F., Sondergaard L., Jørgensen T.H., Lopez-Aguilera J., Kodali S., Leon M. (2020). Clinical impact of conduction disturbances in transcatheter aortic valve replacement recipients: A systematic review and meta-analysis. Eur. Heart J..

[22] Wang J., Liu S., Han X., Chen Y., Chen H., Wan Z., Song B. (2022). Prognostic Outcome of New-Onset Left Bundle Branch Block After Transcatheter Aortic Valve Replacement in Patients with Aortic Stenosis: A Systematic Review and Meta-Analysis. Front. Cardiovasc. Med..

[23] Galeotti L., van Dam P.M., Loring Z., Chan D., Strauss D.G. (2013). Evaluating strict and conventional left bundle branch block criteria using electrocardiographic simulations. Europace.

[24] Massoullié G., Souteyrand G., Ploux S., Combaret N., Mondoly P., Pereira B., Amabile N., Irles D., Mansourati J., Mechulan A. (2025). Added Diagnostic Value of Electrophysiological Study in New Onset Left Bundle Branch Block After Transcatheter Aortic Valve Implantation. Can. J. Cardiol..

[25] Junquera L., Freitas-Ferraz A.B., Padrón R., Silva I., Nunes Ferreira-Neto A., Guimaraes L., Mohammadi S., Morís C., Philippon F., Rodés-Cabau J. (2020). Intraprocedural high-degree atrioventricular block or complete heart block in transcatheter aortic valve replacement recipients with no prior intraventricular conduction disturbances. Catheter. Cardiovasc. Interv..

[26] Bagur R., Rodés-Cabau J., Gurvitch R., Dumont É., Velianou J.L., Manazzoni J., Toggweiler S., Cheung A., Ye J., Natarajan M.K. (2012). Need for Permanent Pacemaker as a Complication of Transcatheter Aortic Valve Implantation and Surgical Aortic Valve Replacement in Elderly Patients with Severe Aortic Stenosis and Similar Baseline Electrocardiographic Findings. JACC Cardiovasc. Interv..

[27] Ozier D., Zivkovic N., Elbaz-Greener G., Singh S.M., Wijeysundera H.C. (2017). Timing of Conduction Abnormalities Leading to Permanent Pacemaker Insertion After Transcatheter Aortic Valve Implantation—A Single-Centre Review. Can. J. Cardiol..

[28] Hamdan A., Guetta V., Klempfner R., Konen E., Raanani E., Glikson M., Goitein O., Segev A., Barbash I., Fefer P. (2015). Inverse Relationship Between Membranous Septal Length and the Risk of Atrioventricular Block in Patients Undergoing Transcatheter Aortic Valve Implantation. JACC Cardiovasc. Interv..

[29] Oestreich B.A., Mbai M., Gurevich S., Nijjar P.S., Adabag S., Bertog S., Kelly R., Garcia S. (2018). Computed tomography (CT) assessment of the membranous septal anatomy prior to transcatheter aortic valve replacement (TAVR) with the balloon-expandable SAPIEN 3 valve. Cardiovasc. Revasc. Med..

[30] Boonyakiatwattana W., Maneesai A., Chaithiraphan V., Jakrapanichakul D., Sakiyalak P., Chunhamaneewat N., Slisatkorn W., Chotinaiwattarakul C., Pongakasira R., Wongpraparut N. (2022). Preprocedural and procedural variables that predict new-onset conduction disturbances after transcatheter aortic valve replacement. BMC Cardiovasc. Disord..

[31] Baraka M., Kamal D., Mostafa A.E. (2024). Depth of implantation in relation to membranous septum as a predictor of conduction disturbances after transcatheter aortic valve implantation. Indian Pacing Electrophysiol. J..

[32] Fujita B., Kütting M., Seiffert M., Scholtz S., Egron S., Prashovikj E., Börgermann J., Schäfer T., Scholtz W., Preuss R. (2016). Calcium distribution patterns of the aortic valve as a risk factor for the need of permanent pacemaker implantation after transcatheter aortic valve implantation. Eur. Heart J. Cardiovasc. Imaging.

[33] Maier O., Piayda K., Afzal S., Polzin A., Westenfeld R., Jung C., Zeus T., Antoch G., Kelm M., Veulemans V. (2021). Computed tomography derived predictors of permanent pacemaker implantation after transcatheter aortic valve replacement: A meta-analysis. Catheter. Cardiovasc. Interv..

[34] De Torres-Alba F., Kaleschke G., Diller G.P., Vormbrock J., Orwat S., Radke R., Reinke F., Fischer D., Reinecke H., Baumgartner H. (2016). Changes in the Pacemaker Rate After Transition From Edwards SAPIEN XT to SAPIEN 3 Transcatheter Aortic Valve Implantation: The Critical Role of Valve Implantation Height. JACC Cardiovasc. Interv..

[35] Tarantini G., Mojoli M., Purita P., Napodano M., D’Onofrio A., Frigo A., Covolo E., Facchin M., Isabella G., Gerosa G. (2015). Unravelling the (arte)fact of increased pacemaker rate with the Edwards SAPIEN 3 valve. EuroIntervention.

[36] Reardon M.J., Chehab B., Smith D., Walton A.S., Worthley S.G., Manoharan G., Sultan I., Yong G., Harrington K., Mahoney P. (2023). 30-Day Clinical Outcomes of a Self-Expanding Transcatheter Aortic Valve: The International PORTICO NG Study. JACC Cardiovasc. Interv..

[37] Möllmann H., Hengstenberg C., Hilker M., Kerber S., Schäfer U., Rudolph T., Linke A., Franz N., Kuntze T., Nef H. (2018). Real-world experience using the ACURATE neo prosthesis: 30-day outcomes of 1000 patients enrolled in the SAVI TF registry. EuroIntervention.

[38] Makkar R.R., Ramana R.K., Gnall E., Ramlawi B., Cheng W., Diamantouros P., Potluri S., Kleinman N., Gupta A., Chakravarty T. (2025). ACURATE neo2 valve versus commercially available transcatheter heart valves in patients with severe aortic stenosis (ACURATE IDE): A multicentre, randomised, controlled, non-inferiority trial. Lancet.

[39] Husser O., Kim W.-K., Pellegrini C., Holzamer A., Walther T., Mayr P.N., Joner M., Kasel A.M., Trenkwalder T., Michel J. (2017). Multicenter Comparison of Novel Self-Expanding Versus Balloon-Expandable Transcatheter Heart Valves. JACC Cardiovasc. Interv..

[40] Costa G., Barbanti M., Rosato S., Seccareccia F., Tarantini G., Fineschi M., Salizzoni S., Valvo R., Tamburino C., Biancari F. (2022). Real-World Multiple Comparison of Transcatheter Aortic Valves: Insights from the Multicenter OBSERVANT II Study. Circ. Cardiovasc. Interv..

[41] Mack M.J., Leon M.B., Thourani V.H., Makkar R., Kodali S.K., Russo M., Kapadia S.R., Malaisrie S.C., Cohen D.J., Pibarot P. (2019). PARTNER 3—Transcatheter Aortic-Valve Replacement with a Balloon-Expandable Valve in Low-Risk Patients. N. Engl. J. Med..

[42] Popma J.J., Deeb G.M., Yakubov S.J., Mumtaz M., Gada H., O’Hair D., Bajwa T., Heiser J.C., Merhi W., Kleiman N.S. (2019). EVOLUT LOW RISK—Transcatheter Aortic-Valve Replacement with a Self-Expanding Valve in Low-Risk Patients. N. Engl. J. Med..

[43] Garcia S., Gada H., Chehab B., Puri R., Chhatriwalla A., Rollefson W., Thourani V. (2024). TCT-920 Early U.S. Commercial Experience with an Intra-Annular, Self-Expandable Valve in High-Risk Patients: 30-Day Results From the Navitor Postapproval Study. JACC.

[44] Pascual I., Almendárez M., Avanzas P., Álvarez R., Arboine L.A., Del Valle R., Hernández-Vaquero D., Alfonso F., Morís C. (2022). Cusp-overlapping TAVI technique with a self-expanding device optimizes implantation depth and reduces permanent pacemaker requirement. Rev. Esp. Cardiol..

[45] Mendiz O.A., Noč M., Fava C.M., Gutiérrez Jaikel L.A., Sztejfman M., Pleskovič A., Gamboa P., Valdivieso L.R., Gada H., Tang G.H.L. (2021). Impact of Cusp-Overlap View for TAVR with Self-Expandable Valves on 30-Day Conduction Disturbances. J. Interv. Cardiol..

[46] Wienemann H., Maier O., Beyer M., Portratz M., Tanaka T., Mauri V., Ernst A., Waldschmidt L., Kuhn E., Bleiziffer S. (2023). Cusp overlap versus standard three-cusp technique for self-expanding Evolut transcatheter aortic valves. EuroIntervention.

[47] Aljabbary T.F., Komatsu I., Ochiai T., Fremes S.E., Ali N., Burke L., Peterson M.D., Fam N.P., Wijeysundera H.C., Radhakrishnan S. (2024). Cusp overlap method for self-expanding transcatheter aortic valve replacement. Catheter. Cardiovasc. Interv..

[48] Ochiai T., Yamanaka F., Shishido K., Moriyama N., Komatsu I., Yokoyama H., Miyashita H., Sato D., Sugiyama Y., Hayashi T. (2023). Impact of High Implantation of Transcatheter Aortic Valve on Subsequent Conduction Disturbances and Coronary Access. JACC Cardiovasc. Interv..

[49] Van Mieghem N.M., Wöhrle J., Hildick-Smith D., Bleiziffer S., Blackman D.J., Abdel-Wahab M., Gerckens U., Linke A., Ince H., Wenaweser P. (2019). Use of a Repositionable and Fully Retrievable Aortic Valve in Routine Clinical Practice: The RESPOND Study and RESPOND Extension Cohort. JACC Cardiovasc. Interv..

[50] Petronio A.S., Sinning J.-M., Van Mieghem N., Zucchelli G., Nickenig G., Bekeredjian R., Bosmans J., Bedogni F., Branny M., Stangl K. (2015). Optimal Implantation Depth and Adherence to Guidelines on Permanent Pacing to Improve the Results of Transcatheter Aortic Valve Replacement with the Medtronic CoreValve System: The CoreValve Prospective, International, Post-Market ADVANCE-II Study. JACC Cardiovasc. Interv..

[51] Jilaihawi H., Zhao Z., Du R., Staniloae C., Saric M., Neuburger P.J., Querijero M., Vainrib A., Hisamoto K., Ibrahim H. (2019). Minimizing Permanent Pacemaker Following Repositionable Self-Expanding Transcatheter Aortic Valve Replacement. JACC Cardiovasc. Interv..

[52] Nazif T.M., Dizon J.M., Hahn R.T., Xu K., Babaliaros V., Douglas P.S., El-Chami M.F., Herrmann H.C., Mack M., Makkar R.R. (2015). Predictors and Clinical Outcomes of Permanent Pacemaker Implantation After Transcatheter Aortic Valve Replacement. JACC Cardiovasc. Interv..

[53] Moriyama N., Sugiyama Y., Miyashita H., Yokoyama H., Yamaguchi M., Ochiai T., Shishido K., Jalanko M., Yamanaka F., Vähäsilta T. (2023). Hemodynamics and Conduction Disturbance After Transcatheter Aortic Valve Implantation with SAPIEN3 Ultra Versus SAPIEN3: The HomoSAPIEN 2 Study. Am. J. Cardiol..

[54] Faurie B., Souteyrand G., Staat P., Godin M., Caussin C., Van Belle E., Mangin L., Meyer P., Dumonteil N., Abdellaoui M. (2019). Left Ventricular Rapid Pacing Via the Valve Delivery Guidewire in Transcatheter Aortic Valve Replacement. JACC Cardiovasc. Interv..

[55] Glikson M., Nielsen J.C., Kronborg M.B., Michowitz Y., Auricchio A., Barbash I.M., Barrabés J.A., Boriani G., Braunschweig F., Brignole M. (2022). ESC/EHRA 2021—Guidelines on cardiac pacing and cardiac resynchronization therapy. EP Europace.

[56] Fournier A., Robert P., Lattuca B., Duflos C., Duroyon M.-M., Macia J.-C., Schmutz L., Steinecker M., Berdeu J.-M., Gandet T. (2025). A Streamline Strategy for Indication and Length of Telemetry Monitoring After TAVR. Catheter. Cardiovasc. Interv..

[57] Siontis K.C., Kara Balla A., Cha Y.-M., Pilgrim T., Sweda R., Roten L., Reichlin T., Friedman P.A., Windecker S., Siontis G.C.M. (2023). Invasive electrophysiological testing to predict and guide permanent pacemaker implantation after transcatheter aortic valve implantation: A meta-analysis. Heart Rhythm O2.

[58] Knecht S., Schaer B., Reichlin T., Spies F., Madaffari A., Vischer A., Fahrni G., Jeger R., Kaiser C., Osswald S. (2020). Electrophysiology Testing to Stratify Patients with Left Bundle Branch Block After Transcatheter Aortic Valve Implantation. J. Am. Heart Assoc..

[59] Meduri C.U., Kereiakes D.J., Rajagopal V., Makkar R.R., O’Hair D., Linke A., Waksman R., Babliaros V., Stoler R.C., Mishkel G.J. (2019). Pacemaker Implantation and Dependency After Transcatheter Aortic Valve Replacement in the REPRISE III Trial. J. Am. Heart Assoc..

[60] Wang X., Xu Y., Zeng L., Tan K., Zhang X., Han X., Xiong T., Zhao Z., Peng Y., Wei J. (2024). Long-term outcomes of left bundle branch area pacing compared with right ventricular pacing in TAVI patients. Heart Rhythm.

[61] Rodés-Cabau J., Urena M., Nombela-Franco L., Amat-Santos I., Kleiman N., Munoz-Garcia A., Atienza F., Serra V., Deyell M.W., Veiga-Fernandez G. (2018). Arrhythmic Burden as Determined by Ambulatory Continuous Cardiac Monitoring in Patients with New-Onset Persistent Left Bundle Branch Block Following Transcatheter Aortic Valve Replacement: The MARE Study. JACC Cardiovasc. Interv..

[62] Natarajan M.K., Sheth T.N., Wijeysundera H.C., Chavarria J., Rodes-Cabau J., Velianou J.L., Radhakrishnan S., Newman T., Smith A., Wong J.A. (2022). Remote ECG monitoring to reduce complications following transcatheter aortic valve implantations: The Redirect TAVI study. Europace.

[63] Zhang Y., Xiong T.-Y., Yang X.-M., Chen D.-F., Li Y.-M., Bao Y., Chen M. (2023). Ambulatory Smartwatch ECG Monitoring among Patients Undergoing Transcatheter Aortic Valve Replacement Early after Discharge: An Observational Study. Rev. Cardiovasc. Med..

[64] Fan J., Dai H., Guo Y., Xu J., Wang L., Jiang J., Lin X., Li C., Zhou D., Li H. (2024). Smartwatch-Detected Arrhythmias in Patients After Transcatheter Aortic Valve Replacement (TAVR): Analysis of the SMART TAVR Trial. J. Med. Internet Res..

[65] Bendandi F., Taglieri N., Ciurlanti L., Mazzapicchi A., Foroni M., Lombardi L., Palermo F., Filice F., Ghetti G., Bruno A.G. (2024). Development and validation of the D-PACE scoring system to predict delayed high-grade conduction disturbances after transcatheter aortic valve implantation. EuroIntervention.

[66] Jia Y., Li Y., Luosang G., Wang J., Peng G., Pu X., Jiang W., Li W., Zhao Z., Peng Y. (2024). Electrocardiogram-based prediction of conduction disturbances after transcatheter aortic valve replacement with convolutional neural network. Eur. Heart J. Digit. Health.

[67] Ueyama H.A., Miyamoto Y., Hashimoto K., Watanabe A., Kolte D., Latib A., Kuno T., Tsugawa Y. (2024). Comparison of Patient Outcomes Between Leadless vs Transvenous Pacemakers Following Transcatheter Aortic Valve Replacement. JACC Cardiovasc. Interv..

